# Synthesis and characterization of zeolites prepared from industrial fly ash

**DOI:** 10.1007/s10661-014-3815-5

**Published:** 2014-05-18

**Authors:** Wojciech Franus, Magdalena Wdowin, Małgorzata Franus

**Affiliations:** 1 Department of Geotechnics, Lublin University of Technology, Lublin, Poland; 2 Mineral and Energy Economy Research Institute of the Polish Academy of Sciences, Kraków, Poland

**Keywords:** Fly ash, Synthesis reactions, Na-X, Na-P1, Sodalite

## Abstract

In this paper, we present the possibility of using fly ash to produce synthetic zeolites. The synthesis class F fly ash from the Stalowa Wola SA heat and power plant was subjected to 24 h hydrothermal reaction with sodium hydroxide. Depending on the reaction conditions, three types of synthetic zeolites were formed: Na-X (20 g fly ash, 0.5 dm^3^ of 3 mol · dm^−3^ NaOH, 75 °C), Na-P1 (20 g fly ash, 0.5 dm^3^ of 3 mol · dm^−3^ NaOH, 95 °C), and sodalite (20 g fly ash, 0.8 dm^3^ of 5 mol · dm^−3^ NaOH + 0.4 dm^3^ of 3 mol · dm^−3^ NaCl, 95 °C). As synthesized materials were characterized to obtain mineral composition (X-ray diffractometry, Scanning electron microscopy-energy dispersive spectrometry), adsorption properties (Brunauer-Emmett-Teller surface area, N_2_ isotherm adsorption/desorption), and ion exchange capacity. The most effective reaction for zeolite preparation was when sodalite was formed and the quantitative content of zeolite from X-ray diffractometry was 90 wt%, compared with 70 wt% for the Na-X and 75 wt% for the Na-P1. Residues from each synthesis reaction were the following: mullite, quartz, and the remains of amorphous aluminosilicate glass. The best zeolitic material as characterized by highest specific surface area was Na-X at almost 166 m^2^ · g^−1^, while for the Na-P1 and sodalite it was 71 and 33 m^2^ · g^−1^, respectively. The ion exchange capacity decreased in the following order: Na-X at 1.8 meq · g^−1^, Na-P1 at 0.72 meq · g^−1^, and sodalite at 0.56 meq · g^−1^. The resulting zeolites are competitive for commercially available materials and are used as ion exchangers in industrial wastewater and soil decontamination.

## Introduction

Combustion and energy production in conventional dust boilers creates two types of wastes: energy slag (furnace) and fly ash. It has been reported that approximately 750 million tonnes of coal fly ash (CFA) has been produced globally, from which only on average only of 25 % is utilized, the rest is disposed as a waste causing yet another environmental concern (Blissett and Rowson 2012). Although composition of CFA is complex and varies greatly (Vassilev and Vassileva 2005), its utilization has been receiving a great deal of attention and coal fly ash is one of the most widely studied pollutants (Mehra et al. 1998). Fly ash storage on heaps or in wet settlers has a significant and costly effect on the environment. Dust must be suppressed and isolated to prevent the migration of different types of pollutants such as heavy metals into the environment (Klojzy-Kaczmarczyk 2003; Chaudhary and Gosh 2013).

Fly ash constitutes 65 % of the total waste production from coal combustion (ECOBA 2009). Currently, less than half is subjected to treatment by re-use (Matsi and Keramidas 1999; Swanepoel and Strydom 2002; Majchrzak-Kuceba 2011; De la Varga et al. 2012; Sumer 2012; Yang et al. 2012), including:

- macro-leveling, soil stabilization
- in mines as backfilling material and material for disposal wells
- ceramics (brick manufacturing)
- the production of building materials (cement and concrete)
- soil fertilization (alkalinization and increased sorption complexes)
- geopolymer construction
- road construction
- CO_2_ sequestration

The suitability of using fly ash is determined mainly by its mineral and chemical composition, which is dependent on the type of combustion coal, furnace, and combustion conditions. Two classes of fly ashes are distinguished based on their chemical components (i.e., SiO_2_ + Al_2_O_3_ + Fe_2_O_3_) and CaO content: class F, a low-calcium ash and class C, which includes high-calcium ash (ASTM C 618–08).

The main mineral component of class F fly ash is aluminosilicate glass accompanied by crystalline mullite, iron oxides (hematite, magnetite), quartz, and unburned carbon fragments. For fly ash containing desulfurization flue gas products, additional small quantities of gypsum/anhydrite, calcite, and calcium were observed (Derkowski 2001; Franus and Wdowin 2011; Franus 2012a).

In the search for new prospective economic uses of fly ash, their similarity to some natural materials such as zeolites was observed in terms of their chemical and mineralogical composition.

Zeolites are microporous, hydrated aluminosilicates of alkali elements, alkaline earth metals, or other cations, which, in their crystal structure, contain numerous channels and chambers of different sizes (in the order of several angstroms). This provides them with a number of sorption, ion exchange, molecular-sieve, and catalyst properties (Ouki and Kavannagh 1997; Panayotova 2003). Because of these properties, they are a widely used mineral resource in many areas of industry, such as agriculture, medicine, chemical technology, environmental protection, and engineering (Payara and Dutta 2003).

Synthetic zeolitic materials can be generated in the chemical reactions of sodium silicate and sodium aluminate and mineral resources (clay minerals, minerals from silica groups) and some are by-products from waste coal circumstantial combustion products (such as fly ash). Using a suitable laboratory methodology, various different zeolitic structures can be obtained including: analcime, chabazite, cancrinite, gmelinite, Na-P1, ZSM-5, ZSM-28, Na-X, Na-Y, philipsite, and sodalite (Querol et al. 1995; Hollman et al. 1999; Yaping et al. 2008; Gatta et al. 2012; Purnomo et al. 2012; Adamczyk and Białecka 2005; Wałek et al. 2008; Ściubidło et al. 2009; Derkowski et al. 2007; Franus and Wdowin 2010; Franus 2012b; Sarbak 2012; Wdowin et al. 2014a, c).

Studies on the transformation of fly ash into zeolites are also important because of the shortage of natural minerals of this type in Poland (Franus and Dudek 1999). The usage of fly ash in zeolitic material synthesis can provide for new development methods and reduce the need for their storage. The properties of zeolitic fly ash products allow for their wide practical application in environmental engineering such as for the removal of contaminants from water and wastewater, drying and gas purification, and regeneration of transformer oil.

## Materials and methods

### Fly ash characterization

The basic substrate in the hydrothermal synthesis of zeolitic material was fly ash from the Stalowa Wola SA heat and power plant, a product of combustion of coal cofired with 10 wt% by weight of biomass.

### Zeolite material synthesis conditions

To obtain a variety of zeolitic materials from fly ash using the synthesis methods proposed by Derkowski et al. (2006) and Franus (2012b), a series of experiments were performed according to the following chemical reaction:

1 $$ \begin{array}{ccc}\hfill \mathrm{fly}\;\mathrm{ash}+x\;\mathrm{mol}\;\mathrm{d}{\mathrm{m}}^{-3}\mathrm{NaOH}\hfill & \hfill \underset{\mathrm{temperature}}{\overset{\mathrm{time}}{\to }}\hfill & \hfill \mathrm{zeolite}+\mathrm{residuum}\hfill \end{array} $$

where *x* is the concentration of NaOH solutions (express as mol) and *x =* 3 or 5 depending on obtained materials.

Changing the synthesis conditions resulted in obtaining three types of zeolites: Na-X, Na-P1, and synthetic sodalite.

For each type of zeolite the following process conditions were applied:

- Synthesis of Na-X phase: 20 g fly ash was mixed with 0.5 dm^3^ NaOH (3 mol · dm^−3^) for 24 h at 75 °C.
- Synthesis of Na-P1 phase: 20 g fly ash was mixed with 0.5 dm^3^ NaOH (3 mol · dm^−3^) for 24 h at 95 °C.
- Synthesis of sodalite phase: 20 g fly ash was mixed with 0.8 dm^3^ NaOH (5 mol · dm^−3^) and 0.4 dm^3^ NaCl (3 mol · dm^−3^) for 24 h at 95 °C.

The resultant zeolitic materials were subjected to mineralogical (X-ray diffractometry (XRD) and scanning electron microscopy-energy dispersive spectrometry (SEM-EDS)), physical (particle size distribution and specific gravity), adsorption (Brunauer-Emmett-Teller (BET)-specific surface area and structure of pores), and cation exchange capacity (CEC) examination.

### Characterization of materials

The chemical composition of the fly ash used for synthesis reactions was determined by XRF method with the use of Philips spectrometer PW 1404 with an X-ray tube equipped with dual Cr-Au anode with a maximum power of 3 kW as the excitation source.

The mineral composition of synthetic zeolites was determined via powder XRD using a Philips X’pert APD diffractometer (PANalytical, Almelo, the Netherlands) with PW 3020 goniometer, Cu lamp, and graphite monochromator from 5 to 65° 2θ. Diffraction data was processed by Philips X’Pert and ClayLab ver. 1.0 software. Mineral phases were identified based on the PCPDFWIN ver. 1.30 database formalized by the Joint Committee on Powder Diffraction Standards—The International Centre for Diffraction Data.

The morphology and chemical composition of the main mineral components of synthetic zeolites in the micro area domain were determined using an FEI Quanta 250 FEG SEM equipped with a chemical composition analysis system based on energy dispersion scattering EDS-EDAX.

- Specific density was tested according to standard PN-EN 1097–7:2008.
- Granulometric analysis was performed using a laser particle analyzer Fritsch GmBH Analysette 22, Idar-Oberstein, Germany, equipped with helium-neon laser, optical system, measuring flow cell for suspensions, and a dispersing unit.

The CEC of the synthetic zeolites was determined on the basis of the amount of Ba^2+^ ions saturated in the sample and desorbed by 1 mol · dm^−3^ MgCl_2_. In the first stage, samples were divided into portions from 33 to 500 mg, mixed with 0.01 dm^3^ of 0.1 mol · dm^−3^ solutions of BaCl_2_, and shaken for 30 min. This was repeated five times. The concentrations of Na, K, Mg, and Ca ions in solution were determined by Varian SpektrAA 880 atomic absorption spectrometer (AAS) and mean CEC values calculated. To desorb the Ba cations, the samples were washed and dried before being treated with 1 mol · dm^−3^ MgCl_2_ solution. The Ba content was measured by AAS. This procedure is recommended by the Association International Pour L’Etude Des Argiles (Derkowski et al. 2006).

The textural properties were investigated by the means of the ASAP 2020 Micromeritics analyzer. The BET-specific surface area as well as pore size and radius are dependent on distribution and were determined based on the shape of the vapor nitrogen adsorption/desorption isotherm at −196.15 ºC. In case elastic sorbent (like coals) it should be use CO_2_ as a sorbate at 24.85 ºC (Zarębska et al. 2012). Prior to analysis, the samples were degassed under strictly controlled conditions at temperature (250 °C, for 24 h) and reduced pressure (10^−3^ hPa).

The specific surface area was determined based on the BET multilayer adsorption theory (Gregg and Sing 1982) at a *p*/*p*_0_ between 0.06 and 0.3 (p and p_0_ are the equilibrium and saturation pressure of nitrogen, respectively). The pore volume (*V*_p_) was determined from the volume of adsorbed nitrogen at pressure *p*/*p*_0_ = 0.98.

Pore diameters (*D*_p_) were calculated according to the formula: *D*_p_ = 4*V*_p_/*S*_BET_, where *S*_BET_ is the BET surface area. The pore volume distribution (*R*_p_) was calculated using a general isotherm equation based on the combination of a modified Kelvin equation and a statistic thickness of the adsorbed film (Wdowin and Gruszecka 2012; Wdowin et al. 2014b).

## Results and discussion

### Characterization of fly ash

The basic chemical oxide composition of fly ash expressed as percentage (%) was as follows: SiO_2_, 53.25; Al_2_O_3_, 26.76 (SiO_2_/Al_2_O_3_, 1.99); Fe_2_O_3_, 5.98; MgO, 2.29; CaO, 2.88; Na_2_O, 0.74; K_2_O, 2.82; TiO_2_, 1.15; P_2_O_5_, 0.47; and SO_3_, 0.48.

**Fig. 1:**
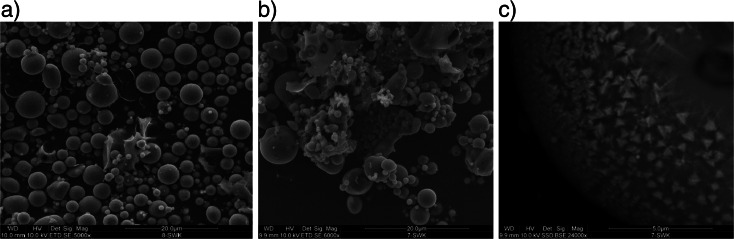
SEM photomicrographs of fly ash from the Stalowa Wola S.A. heat and power plant. **a** spherulites of aluminosilicate glass. **b** glass spherulites fragments of unburned carbon, **c** iron oxide inclusions onto aluminosilicate sphere

The scanning electron microscopy showed that the dominant morphological forms occurring in the studied fly ash are spheres of amorphous aluminosilicate glass. Their size varies from a few micrometers to 0.2 mm. These spheres occur individually or form intergrowths. Grains exist in a few percent of unburned carbonaceous matter. They have different shapes and are generally porous, with pore maximum size of a few micrometers. X-ray diffractometry (XRD) results indicated the presence of cryptocrystalline aggregates composed of mullite and a small amount of quartz and iron oxides. Iron oxides were observed on the outer surface of spherical forms of aluminosilicate glass in the form of very fine magnetite-hematite inclusions (Fig. 1). The bulk density of the tested ash was 2.28 g · cm^−3^.

Analysis of the fly ash particle size showed a bimodal particle size distribution with a first maximum in the range of approximately 20 μm and the second at c.a. 150 μm. These maxima are distinct and characteristical for a low diversity of grains. Textural analysis showed that the fly ash has a very low specific Brunauer-Emmett-Teller (BET) surface area of 12 m^2^ · g^−1^ and low ion exchange capacity of 0.10 meq · g^−1^.

### Characterization of obtained zeolites

**Fig. 2:**
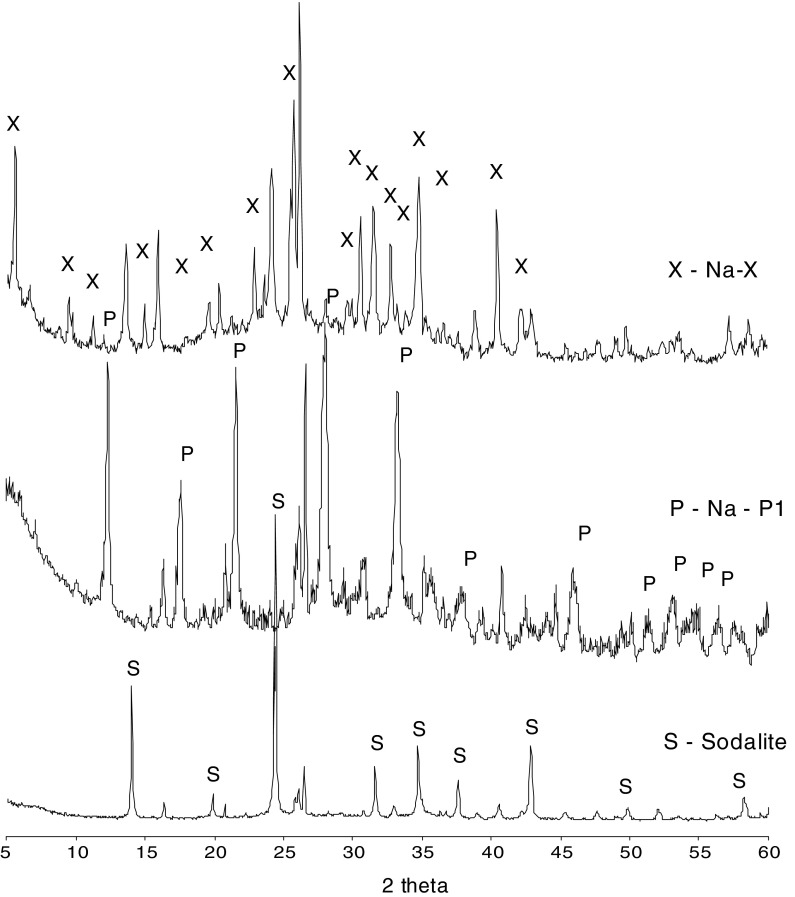
Mineral composition of the zeolitic material (Na-X, Na-P1, Sodalite)

For each proposed synthesis condition, zeolitic materials were obtained in the reaction products as the main mineral phases. Diffractograms of mineral composition for different types of zeolites are shown in Fig. 2. The presence of a Na-X zeolitic phase in the reaction products was determined based on the main d-spacing d_hkl_ = 14.47, 3.81, 5.73, 8.85, 4.42, 7.54, 4.81, and 3.94 Å. The quantitative zeolite content as calculated from XRD analysis was 70 wt%. The presence of a Na-P1 zeolitic phase in the reaction products was determined based on the main d-spacing d_hkl_ = 7.10, 5.01, 4.10, and 3.18 Å. The quantitative zeolite content calculated from XRD was 75 wt%. The presence of a sodalite phase in the reaction products was determined based on the main d-spacing d_hkl_ = 6.29, 3.62, and 2.09 Å. The quantitative content of the zeolitic phase from XRD analysis was 90 wt%. Residues from the synthesis reactions included mullite, quartz, and amorphous aluminosilicate glass.

The particle size distribution of all three types of zeolitic materials is very similar, and is characterized by a monomodal distribution with a maximum in the range of 26 to 30 μm.

**Fig. 3:**
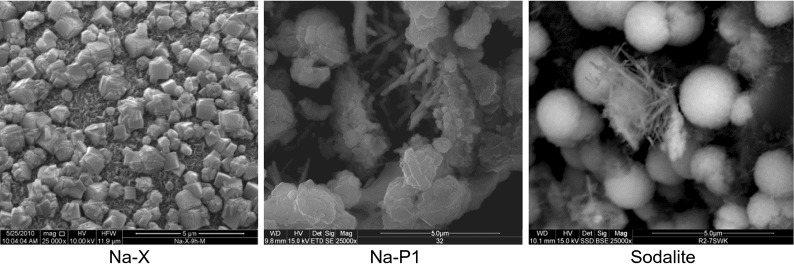
SEM microphotographs of obtained zeolitic materials. **a** Na-X, **b** Na-P1, **c** sodalite

Observations of the morphology of zeolite crystals and their habit allowed the identification of individual representatives of this group of minerals. Observation of crystal habits is useful in communicating what specimens of a particular mineral will often look like, what will allow the distinguishing of this zeolites. Depending on the zeolite type, various of crystals were observed. SEM observations (Fig. 3) of grain shapes showed that the zeolitic material type Na-X consists of isometric and octahedral crystals approximately 1–2 μm in size, often intergrown with one another. Zeolites with such crystals (growth of crystals parallel in three dimensions) are characteristic for the faujasite group of zeolites. Zeolite Na-P1 forms lamellar aggregates about plate-like habits with a length of 2–3 μm. Such habits are characteristic for gismondite group of zeolites. Sodalite occurs in spherical form (like desert roses) with a diameter of 2–3 μm. The habit of sodalite is isometric (similar as in Na-X).

Microprobe chemical analysis (SEM-EDS) of the Na-X showed that sodium is the main exchange cation in the structure, balancing the charge of the aluminosilicate lattice. The mean ratios of the individual cations derived from the microchemical point analysis EDS are as follows: Na + K/Si = 0.55, Na + K + Ca + Mg/Si = 0.60, Na + K/Si = 0.65, Na + K + Ca + Mg/Si = 0.79, and Si/Al = 1.12. For Na-P1, sodium is also the main exchange cation in the structure balancing the charge of the aluminosilicate lattice. The mean ratios of individual cations derived from the microchemical point analysis EDS are as follows: Na + K + Ca + Mg/Si = 0.44, and Si/Al = 1.42. Similarly, for the sodalite, sodium is the main exchange cation in the structure balancing the charge of the aluminosilicate lattice. The mean ratios of the individual cations derived from the microchemical analysis point EDS are as follows: Na + K + Ca + Mg/Si = 1.12 and Si/Al = 1.21.

The ion exchange capacity of Na-X was 1.8 meq · g^−1^, for the Na-P1 it was 0.72 meq · g^−1^ and 0.56 meq · g^−1^ for the sodalite.

**Fig. 4:**
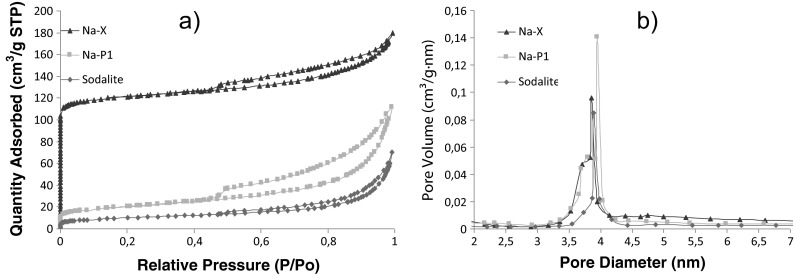
**a** Isotherms adsorption/desorption of N_2_ (Na-X, Na-P1, sodalite). **b** the distribution of the pore structure (Na-X, Na-P1, sodalite)

The resulting isotherms for the analyzed zeolites are represented by I Type isotherms according to the IUPAC classification. This indicates that the tested zeolitic materials are mesoporous, in which the predominant pore size is close to the micropore size (Na-P1 and sodalite). However, the Na-X zeolitic material can be classified as microporous. Hysteresis loops can be classified as the H4 Type, characterized by the presence of narrow slit-like pores (Fig. 4a).

The basic textural parameters obtained for the zeolitic materials are shown in Table 1. Analysis of the Na-X textural results shows a significant increase in surface area in relation to the fly ash from which it originated. The BET-specific surface area in relation to the fly ash increased from approximately 10 to 166 m^2^ · g^−1^ for Na-X. Practically, all textural parameters (micropore volume and surface, mesopore volume and BET surface area) show a clear upward trend in relation to the textural fly ash data. A similar situation can be observed for other zeolitic materials, where the surface area of the Na-P1 was 71 m^2^ · g^−1^ and for sodalite, 33 m^2^ · g^−1^.

**Table 1:** Textural parameters of obtained zeolitic materials

Material	S_BET_ [m^2^ · g^−1^]	V_mic_ [cm^3^ · g^−1^]	S_mic_ [m^2^ · g^−1^]	V_mes_ [cm^3^ · g^−1^]		S_mes_ [m^2^ · g^−1^]		Average pore diameter [nm]	
				Ads.	Des.	Ads.	Des.	Ads.	Des.
Na-X	166	0.054	116	0.094	0.097	52.126	64.224	8.828	7.451
Na-P1	71	0.006	13	0.162	0.171	52.406	75.893	12.397	9.027
Sodalite	33	0.002	5	0.100	0.096	29.627	33.155	13.613	11.693

The pore size distribution ranges from 1.7 to 100 nm based on the Barrett-Joyner-Halenda equation shown in Fig. 4b. For Na-X, pores in the range of 3.3 to 4.7 nm dominated with a maximum of approximately 3.8 nm being observed. The average pore diameter determined based on the desorption of nitrogen is approximately 7.4 nm. The Na-P1 pore size distribution indicates the dominance of pores at a maximum of approximately 3.8 nm. The average pore diameter is approximately 9.0 nm. The sodalite pore size distribution shows the predominance of pores in the range of 12 to 22 nm. The average pore diameter for this material is 11.6 nm.

Described synthesis of zeolitic materials helps to use a major waste material such as fly ash. Besides, presented resulting zeolites have similar surface parameters and CEC to the commercial available synthetic zeolitic products. Such results allow the successful use of this material in purification of environmental pollutions. Additionally, production of zeolite from fly ash is cost effective in comparison to zeolites synthesized by using other materials i.e., from perlite (Pichór et al. 2014), kaolinite (Novembre et al. 2011), and illite-smectite (Baccouche et al. 1998). For example, production of Na-X and hydroksysodalite from metakaolinite requires high calcination temperature of kaolinite (650 °C) and further 68 °C for hydrothermal synthesis (Novembre et al. 2011). A pure zeolite from the faujasite group is obtained from synthesis reaction with perlite (Christidis and Papantoni 2008; Pichór et al. 2014). Another advantage of described zeolites is their content in the final solids where Na-X was 70 wt%, Na-P1—75 wt%, and sodalite 90 wt%, respectively. It is a much better result in comparison to zeolites obtained from illite-smectite were their content in the final products was 47–58 wt% for Na-P1 and 76 % of sodalite, respectively (Baccouche et al. 1998).

### Potential applications

The resulting zeolitic materials can be used to remove a number of pollutants in environmental technologies.

Zeolitic materials formed from fly ash may constitute a high-grade mineral sorbent for the removal of ammonium ions from aqueous solutions (Wang et al. 1998; Franus and Wdowin 2010; Niu et al. 2012).

Synthetic zeolites (e.g., Na-chabazite, Na-P1, F, KM, sodalite and analcime, and mixture of 4A-X, Na-X), obtained by the transformation of fly ash, can be a valuable absorbent material for the gaseous forms of NH_3_ and Hg as well as CO_2_, SO_2_ (Querol et al. 2002; Morency et al. 2002; Wdowin et al. 2012; Wdowin et al. 2014c).

Zeolitic material rich in zeolites of type Na-X and Na-P1 can also be used in the refinery and petrochemical industry in adsorption refining and could compete with commercial products such as attapulgite, Al_2_O_3_, and silica gel, sorbents that remove acidic compounds from used transformer oil and compounds that impair color (which eliminates the possibility of their use). The use of zeolites as oils for capturing this type of pollution ensures that these oils can be reused (Beran and Rutkowski 1994; Derkowski et al. 2006).

Another promising direction is the use of zeolitic materials to remove elevated contents of radium isotopes ^226^Ra and ^228^Ra from mines and underground drinking water and galvanizing wastewater with heavy metals. Materials rich in Na-P1 zeolite can be used successfully to remove these impurities (Franus 2012b; Chałupnik et al. 2013).

Major perspective applications of the synthetic zeolites are based on their use as high ion exchangers in industrial wastewater and soil decontamination. For example, in zinc ions removal, they were applied both zeolite Na-X synthesized from fly ash (de Izidoro et al. 2013) as well as from kaolin (Ismael 2010). In both cases, the results have shown that investigated zeolites could be a beneficial product, which will be used in the future as ion exchangers in removal of zinc ions from wastewaters, because zinc is more preferentially adsorbed on zeolites than other heavy metals (cadmium) occurring in wastewaters (de Izidoro et al. 2013).

## Discussion and conclusions

For improved environmental protection, it is becoming increasingly important to find new, promising ways to use wastes such as fly ash.

The synthesis of zeolites from this type of waste material is justified as the waste, whose disposal constitutes an environmental/ecological problem, is consumed. Furthermore, a material with advantageous ion exchange and adsorption properties is formed, for use in environmental technologies such as the removal of heavy metals and ammonium ions from wastewaters and sewage, radioactive elements from mine water, and the drying and cleaning of industrial gases, as well as hydrocarbon sorption.

The aim of this study was to present the possibility of using fly ash for the production of synthetic zeolites. In comparison with the natural zeolites, synthetic zeolites may be a more promising mineral sorbent for use in environmental engineering and protection because natural zeolites have a well-defined channel size for the removal of a limited size of molecular contaminant types from water or air.

The synthesis reactions that were carried out have shown that, depending on the synthesis conditions of the process (i.e., NaOH concentration and reaction temperature), several types of zeolites with different channel system sizes can be obtained. From laboratory work, we obtained three types of synthetic zeolite materials: Na-X (20 g fly ash, 0.5 dm^3^ of 3 mol · dm^−3^ NaOH, 75 °C), Na-P1 (20 g fly ash 0.5 dm^3^ of 3 mol · dm^−3^ NaOH, 95 °C), and sodalite (20 g fly ash 0.8 dm^3^ of 5 mol · dm^−3^ NaOH and 0.4 dm^3^ of 3 mol · dm^−3^ NaCl, 95 °C).

We compared the properties and characteristics of the zeolitic material to assess which synthesis reaction was most effective. The zeolites obtained seem to be most promising in their practical application as a mineral sorbent.

Mineralogical analysis indicated that this type of laboratory scale reaction yielded a high percentage (>70 wt%) of a particular zeolite in relation to the residue. The most effective reaction for obtaining a zeolite from fly ash was for sodalite, where the quantitative content of this zeolite calculated by XRD was 90 wt%. The least effective synthesis reaction was for Na-X.

Analysis of the textural parameters showed that Na-X displayed the best properties with a surface area of 166 m^2^ · g^−1^, which was much higher than that of Na-P1 (71 m^2^ · g^−1^) and sodalite (33 m^2^ · g^−1^). A similar relationship existed for the ion exchange capacities 1.8, 0.72, and 0.56 meq · g^−1^ for Na-X, Na-P1, and sodalite, respectively.

In conclusion, it is evident that the most efficient synthesis reaction is that for sodalite and the most preferred in practical applications for decontamination is the Na-X zeolite.

The obtained zeolite material can be used widely to remove impurities in the form of heavy metals, radioactive elements, ammonium ions from water and wastewater as well as a number of gases such as sorbent CO_2_, SO_2,_ or mercury.
